# Dietary Chlorogenic Acid Supplementation Alleviates Heat Stress-Induced Intestinal Oxidative Damage by Activating Nrf2 Signaling in Rabbits

**DOI:** 10.3390/antiox15010002

**Published:** 2025-12-19

**Authors:** Jiali Chen, Rongmei Ji, Fuchang Li, Lei Liu

**Affiliations:** Key Laboratory of Efficient Utilization of Non-Grain Feed Resources (Co-Construction by Ministry and Province), Ministry of Agriculture and Rural Affairs, Shandong Provincial Key Laboratory of Animal Nutrition and Efficient Feeding, Department of Animal Science and Technology, Shandong Agricultural University, Panhe Street 7, Tai’an 271017, China; jlchen@sdau.edu.cn (J.C.); 17861511020@163.com (R.J.); chlf@sdau.edu.cn (F.L.)

**Keywords:** heat stress, rabbit, chlorogenic acid, intestine oxidative damage, antioxidant capacity

## Abstract

Heat stress (HS) significantly threatens the sustainability of the rabbit industry, primarily by inducing oxidative damage to the intestine, which compromises both the health and productivity of rabbits. Chlorogenic acid (CGA) belongs to a major class of natural polyphenols and possesses significant antioxidant properties. This study aimed to elucidate the protective effects and underlying mechanisms of CGA against HS-induced intestinal damage in rabbits. In vivo, compared with the HS group, CGA significantly elevated serum CAT and SOD activities (*p* < 0.05), as well as reduced serum MDA and jejunal HSP70 levels (*p* < 0.05) in HS-challenged rabbits. In addition, CGA reversed HS-induced downregulation of antioxidant genes (*HO-1*, *SOD1*) and upregulation of apoptosis-related genes (*Bax*, *caspase-3*) (*p* < 0.05). In vitro, CGA significantly suppressed HS-induced intestinal epithelial cell apoptosis, ROS overproduction, and tight junction protein (*occludin*, *ZO-1*) downregulation (*p* < 0.05) by activating Nrf2 signaling. Specific inhibition of Nrf2 significantly abolished CGA’s protective effects. These results strongly suggest that CGA alleviates HS-induced intestinal oxidative damage and maintains barrier integrity via Nrf2 signaling. This finding offers a safe nutritional intervention to enhance HS resistance and growth performance in rabbits, addressing a key constraint to the sustainability of the rabbit industry amid global warming.

## 1. Introduction

Global warming is progressively intensifying, particularly during the hot and humid summer months, presenting a substantial challenge to the livestock industry [1,2]. In this context, rabbits are particularly vulnerable due to their physiological limitations, such as the absence of sweat glands and their fur-covered bodies, which make them highly susceptible to elevated environmental temperatures [3]. As a result, heat stress (HS) has become a significant concern in global rabbit production [2,4]. One common symptom of HS in rabbits is intestinal damage, which is often accompanied by physiological dysfunction and metabolic disorders, ultimately leading to growth retardation [5,6]. The pathogenesis of this intestinal injury is closely linked to oxidative stress. Under HS conditions, reactive oxygen species (ROS) production disrupts intestinal epithelial cells (IECs) redox balance, impairs tight junction proteins (TJPs) like claudin-1, occludin, and ZO-1, thereby increasing intestinal permeability [7]. The impairment of the intestinal barrier has been identified as a key pathological factor contributing to the decline in rabbit health under HS conditions. Therefore, identifying safe and effective strategies to protect the intestine has emerged as a research priority in the fields of animal nutrition and physiology. Consequently, there is an urgent need for potent and safe interventions to maintain intestinal health in this critical context.

In recent years, plant-derived bioactive compounds have gained significant attention as potential alternatives to synthetic antioxidants and growth promoters, partly due to their remarkable antioxidant and gut-modulatory properties, as well as their high safety profile and environmental compatibility [8]. Chlorogenic acid (CGA), a prominent polyphenolic compound found in various plants such as coffee beans, green tea, and sunflower seeds, has been extensively studied for its diverse biological activities [9,10,11]. Extensive evidence indicated that CGA could effectively scavenge free radicals, inhibit lipid peroxidation, and modulate the expression of antioxidant enzymes to mitigate oxidative stress in various cells and tissues [12]. Chen et al. [13] demonstrated that dietary supplementation with 300 and 600 mg/kg CGA suppressed acute HS-induced reductions in antioxidant enzyme activities in young hens, with 600 mg/kg showing the most beneficial effects. Shaukat et al. [14] further showed that supplementing 600 mg/kg CGA in the diets downregulated the expression of heat shock proteins and upregulated the levels of TJPs (including occludin, ZO-1, and claudin-1) in HS-exposed broilers. Notably, our previous study investigated the effects of dietary CGA supplementation at doses of 400, 800, and 1600 mg/kg, and demonstrated that dietary supplementation with 800 mg/kg CGA effectively improved the growth performance of weaned rabbits by enhancing intestinal structural integrity, modulating gut microbiota homeostasis, and alleviating intestinal inflammation and oxidative stress [15]. More recently, we demonstrated that 800 mg/kg CGA could mitigate HS-induced liver damage in rabbits by activating the Nrf2/HO-1 antioxidant pathway, inhibiting hepatic apoptosis, and reversing HS-induced declines in growth performance (restoring ADG and F/G) [16]. These findings collectively confirm that 800 mg/kg CGA exerts beneficial effects on improving growth performance and maintaining the health of rabbits under different stress conditions. However, whether the growth-promoting effect of CGA on HS-challenged rabbits is mediated through the alleviation of intestinal damage, one of the main pathological manifestations of HS in rabbits, remains unclear. Furthermore, the underlying molecular mechanisms warrant further investigation.

Based on the above, we therefore hypothesized that dietary supplementation with CGA may alleviate HS-induced intestinal damage in rabbits. This protective effect was likely mediated through the enhancement of the antioxidant defense system to mitigate oxidative stress, consequently leading to the preservation of intestinal barrier integrity. Therefore, the primary objectives of the present study were to evaluate the protective effects of dietary CGA supplementation on HS-induced intestinal barrier dysfunction and explore the underlying molecular mechanisms in rabbits.

## 2. Materials and Methods

### 2.1. Reagents

Chlorogenic acid (Cat. No. C3878; purity ≥ 95%) was obtained from Sigma-Aldrich (Shanghai, China). DMEM/F12 medium (Dulbecco’s Modified Eagle’s Medium/Ham’s F-12 Nutrient Mixture) and fetal bovine serum were sourced from HyClone (Logan, UT, USA). ML385 was purchased from MedChemExpress (Monmouth Junction, NJ, USA).

### 2.2. Animal Management

The study received ethical approval from the Experimental Animal Welfare and Ethical Committee of Institute of Animal Science of Shandong Agricultural University (SDAUA-2021-050). A total of 120 newly weaned New Zealand rabbits (60 males and 60 females), averaging 1.04 ± 0.11 kg in initial body weight (BW), were used in this study. Seven days after the pre-test, they were randomly assigned to one of three dietary treatments in a completely randomized design. Each treatment contained 20 replicate cages, with two rabbits housed per cage. The treatments consisted of: control group (CON, rabbits were housed at 25 ± 1 °C, relative humidity was 34 ± 1.5% and fed a basal diet), heat stress group (HS, rabbits were housed at 35 ± 1 °C, relative humidity was 44 ± 1.9% and fed a basal diet), and HS + CGA group (rabbits were housed at 35 ± 1 °C, relative humidity was 44 ± 1.9% and fed a basal diet supplemented with 800 mg/kg CGA [50% purity, provided by Chengdu Hengfeng Tiancheng Technology], Chengdu, China). The dietary supplementation level of CGA was based on our previous findings [15]. Rabbits were housed in an environmentally controlled facility, with free access to food and water throughout the experiment. The treatment duration was 28 days. The basal diet was formulated to meet the nutrient requirements outlined in the Nutrition Requirements of Meat Rabbit (NY/T 4049-2021). The ingredient composition and nutrient levels are detailed in Table S1.

At the end of the trial, eight rabbits were randomly selected from each group for sampling. Blood was collected from the marginal ear vein and centrifuged (3000× *g*, 10 min, 4 °C) to separate the serum, which was stored at −20 °C for further analysis. Following blood collection, the rabbits were sacrificed by cervical dislocation, and the abdomen was immediately opened to collect the jejunal samples. A segment (approximately 5 cm) of the mid-jejunum was excised and sectioned. The portions were then snap-frozen in liquid nitrogen and stored at −80 °C for future analysis.

### 2.3. Isolation and Culture of Rabbit IECs

Primary IECs were isolated from newborn rabbits according to the method described by Fan et al. [17]. Briefly, after euthanizing these rabbits with carbon dioxide inhalation, the small intestine was immediately isolated and placed in a dish containing PBS. A 2 cm segments of the middle intestine was separated, and the mesentery was gently removed with tweezers. The intestinal segments were opened, washed three times with PBS and serum-free DMEM-F12 (containing 300 μg/mL penicillin and 0.3 g/L streptomycin), and then incubated for 20 min in a 37 °C water bath with neutral protease I (0.1 mg/mL) and collagenase (0.4 mg/mL). After digestion, the solution was gently pipetted 100 times, and DMEM-F12 (with 2.5% fetal bovine serum) and 2% sorbitol were added to halt digestion. The solution was centrifuged at 1200× *g* for 5 min, and the supernatant was discarded. The pellet was resuspended in complete medium (10% fetal bovine serum + 1% antibiotics) and filtered through a 70 μm cell strainer. Primary IECs were cultured in DMEM-F12 medium for 24 h. Cell viability was determined using a CCK-8 kit, with a measured viability of 93.26% (n = 6). Following confirmation of satisfactory cell viability, fresh DMEM-F12 medium was added, and the cells were treated with various reagents and harvested at specified time points for further analysis.

### 2.4. Biochemical Analysis

Tissues or cells were lysed by homogenization in ice-cold RIPA buffer. Jejunal HSP70 levels were determined using a commercial rabbit-specific ELISA kit (Beijing Winter Song Boye Biotechnology Co., Ltd., Beijing, China) with a spectrophotometer, following the manufacturer’s instructions. Oxidative stress markers, including malondialdehyde (MDA), catalase (CAT), superoxide dismutase (SOD), glutathione peroxidase (GSH-Px), and total antioxidant capacity (T-AOC), were measured using commercial kits (Nanjing Jiancheng Institute of Bioengineering, Nanjing, China) according to the manufacturer’s protocols.

### 2.5. Quantitative Real-Time PCR

Total RNA was isolated from the jejunal tissues and primary IECs using the TRIzol kit (Invitrogen Inc., Carlsbad, CA, USA). Real-time quantitative PCR was performed to analyze the expression levels of glutathione peroxidase 1 (*GPx1*), NAD(P)H quinone oxidoreductase-1 (*NQO1*), Superoxide dismutase 1 (*SOD1*), *SOD2*, heme-oxygenase 1 (*HO-1*), cysteinyl aspartate-specific proteinase-3 (*caspase-3*), *Fas*, B-cell lymphoma 2 (*Bcl-2*), Bcl-2-associated X protein (*Bax*), *occludin*, *claudin-1* and zonula occludens 1 (*ZO-1*) as previously described [16]. Gene expression levels were normalized to glyceraldehyde-3-phosphate dehydrogenase (*GAPDH*) and calculated using the 2^−ΔΔCt^ method. The primer sequences are provided in Table S2.

### 2.6. Flow Cytometric Assays

Cellular apoptosis was evaluated by Annexin V-fluorescein isothiocyanate (FITC)/Alexa Fluor 647 and propidium iodide (PI) (Biolegend, Cat. Nos. 640914 and 610912, San Diego, CA, USA) double staining, following the manufacturer’s guidelines. Briefly, cells were harvested and resuspended in 1× binding buffer at a concentration of 100 μL per 10^6^ cells. Next, 2 μL of Annexin V was added to each sample, followed by incubation on ice in the dark for 20 min. Subsequently, 400 μL of 1× binding buffer and 1 μL of PI (1 mg/mL) were added to the reaction tubes and mixed thoroughly. Apoptotic cells were immediately detected via flow cytometry (BD Accuri C6, BD Biosciences, Franklin Lakes, NJ, USA) and analyzed using FlowJo software (ModFit LT 5.0).

### 2.7. Immunofluorescence Assays

The cellular distribution of the TJP claudin-1 was examined via immunofluorescence analysis, as described previously [18]. Briefly, cultured cells were fixed with 4% paraformaldehyde for 30 min, followed by permeabilization with 0.5% Triton X-100. After sequential incubation with primary and secondary antibodies, DAPI was added for nuclear fluorescence labeling. Images were acquired using a laser scanning confocal microscope (Nikon, Tokyo, Japan).

### 2.8. Intracellular ROS Assays

ROS levels in rabbit IECs were determined using 2′,7′-dichloro dihydrofluorescein diacetate (DCFH-DA), following the manufacturer’s instructions. After removing the medium, cells were incubated with 1 mL of DCFH-DA working solution (prepared in serum-free medium per kit instructions) at 37 °C for 30 min. The cells were then washed three times with ice-cold serum-free medium while gently shaking to remove any residual extracellular probe. Fluorescent images were immediately captured using a fluorescence microscope (Nikon Ts2R-FL, Tokyo, Japan) equipped with an FITC filter (excitation: 488 nm; emission: 525 nm). All images were obtained under consistent parameters, and fluorescence intensity was quantified using ImageJ software (v1.54g).

### 2.9. Western Blotting Analysis

Western blotting was performed as previously described [18]. Proteins were extracted from primary rabbit IECs using RIPA lysis buffer, and protein concentrations were determined using a BCA protein assay kit. Equal amounts of protein (20–30 μg per lane) were separated by SDS-PAGE and electrophoretically transferred to PVDF membranes using a wet transfer system. Membranes were blocked with 5% BSA in 0.1% TBST for 1 h at room temperature, then incubated overnight at 4 °C with the corresponding primary antibodies. After incubation with appropriate HRP-conjugated secondary antibodies, protein bands were detected by chemiluminescence using a commercial imaging system (Vilber Bio Imaging, Paris, France), and band intensity was quantified by densitometric analysis.

### 2.10. Statistical Analysis

Data from the in vivo and in vitro trials were analyzed by one-way analysis of variance (ANOVA) followed by Tukey’s multiple range test (to identify significant differences among treatments) using SAS 9.4 software (SAS Inst. Inc., Cary, NC, USA). Results are presented as mean ± standard error of the mean (SEM). Statistical significance was defined as *p* < 0.05.

## 3. Results

### 3.1. Impacts of CGA on the Serum Redox Status of HS-Expressed Rabbits (In Vivo)

HS significantly induced oxidative stress in rabbits, as evidenced by increased (*p* < 0.05) MDA levels and decreased (*p* < 0.05) the activity of antioxidant enzymes, such as CAT and SOD, in serum (Table 1). However, CGA supplementation elevated (*p* < 0.05) the activity of antioxidant enzymes and significantly reduced (*p* < 0.05) serum MDA levels in HS-exposed rabbits.

### 3.2. Impacts of CGA on HSP70 Level, and Antioxidant-Related and Apoptosis-Related Genes Expressions in the Jejunum of HS-Challenged Rabbits (In Vivo)

**Figure 1:**
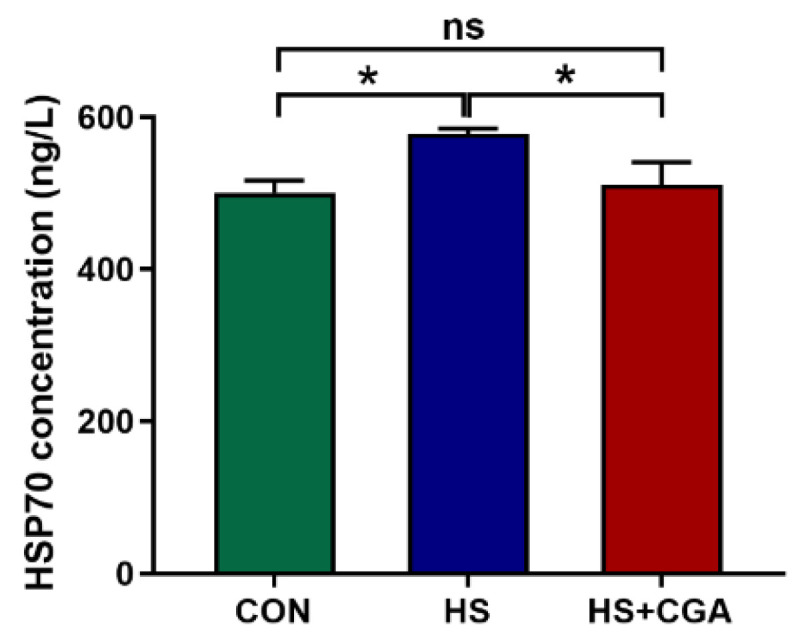
Effects of chlorogenic acid (CGA) on jejunal heat shock protein 70 (HSP70) levels of rabbits under heat stress (HS). CON, rabbits fed a basal diet and housed at optimum temperature; HS, rabbits fed a basal diet and exposed to HS; HS+CGA, rabbits fed a basal diet supplemented with 800 mg/kg CGA and exposed to HS. * *p* < 0.05; ^ns^ *p* > 0.05. n = 8.

**Figure 2:**
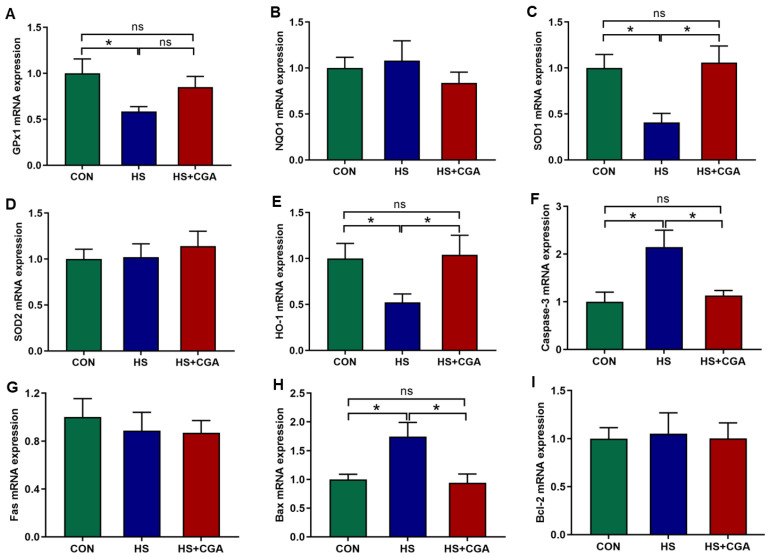
Effects of chlorogenic acid (CGA) on the expressions of antioxidant-related (**A**–**E**) and apoptosis-related (**F**–**I**) genes in the jejunum of rabbits under heat stress (HS). *NQO1*, NAD(P)H quinone oxidoreductase-1; *GPx1*, glutathione peroxidase 1; *SOD*, Superoxide dismutase; *HO-1*, Heme-oxygenase 1; *Bcl-2*, B-cell lymphoma 2; *Bax*, Bcl-2-associated X protein. CON, rabbits fed a basal diet and housed at optimum temperature; HS, rabbits fed a basal diet and exposed to HS; HS+CGA, rabbits fed a basal diet supplemented with 800 mg/kg CGA and exposed to HS. * *p* < 0.05; ^ns^ *p* > 0.05.

As shown in Figure 1, the HS group exhibited significantly higher (*p* < 0.05) jejunal HSP70 levels compared to the CON group. Notably, pretreatment with CGA markedly reduced (*p* < 0.05) the elevated jejunal HSP70 levels induced by HS. Additionally, as shown in Figure 2, HS significantly decreased (*p* < 0.05) the mRNA expression levels of antioxidant-related molecules (*HO-1*, *SOD1* and *GPx1*), while increasing (*p* < 0.05) the mRNA expression levels of apoptosis-related molecules (*Bax* and *caspase-3*) relative to the CON group. However, supplementation with CGA effectively reversed (*p* < 0.05) the HS-induced alterations in the mRNA expression of these molecules in rabbits.

### 3.3. Cell Viability (In Vitro)

**Figure 3:**
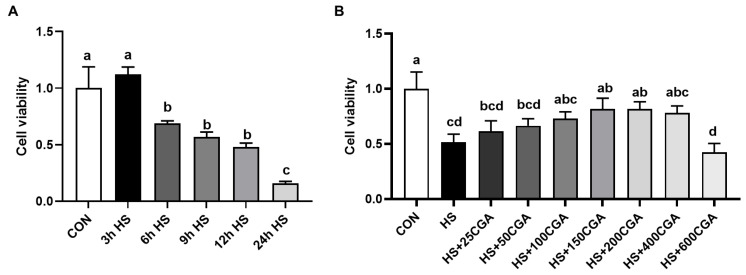
Cell viability. (**A**) Effects of heat stress (HS) at 43 °C on the cell viability of rabbit IECs; (**B**) Effects of different dosages of chlorogenic acid (CGA) on cell viability of IECs under heat stress. ^a,b,c,d^ Means with different letters are significant different (*p* < 0.05). n = 8.

We further examined the protective effect of CGA on HS-induced oxidative stress and injury in rabbit IECs. Cell viability was assessed using a CCK-8 assay. As shown in Figure 3A, cells were exposed to a high temperature of 43 °C for different durations (0, 3, 6, 9, 12, and 24 h). The results indicated that cell viability significantly decreased (*p* < 0.05) after 6 h of HS exposure, dropping to approximately 60%, which met the criteria for establishing an HS model. Therefore, a 6 h exposure at 43 °C was selected for subsequent experiments. To determine the appropriate concentration of CGA to mitigate HS-induced damage, cells were treated with various doses of CGA (0, 25, 50, 100, 150, 200, 400, and 600 μM) while being simultaneously exposed to 43 °C for 6 h (Figure 3B). Under HS conditions, the addition of 150 μM and 200 μM CGA resulted in the most pronounced increase in cell viability. Thus, further studies were conducted to evaluate the effects of these two concentrations (150 and 200 μM) on cell apoptosis.

### 3.4. CGA Alleviates HS-Induced Cell Apoptosis in Rabbit IECs (In Vitro)

**Figure 4:**
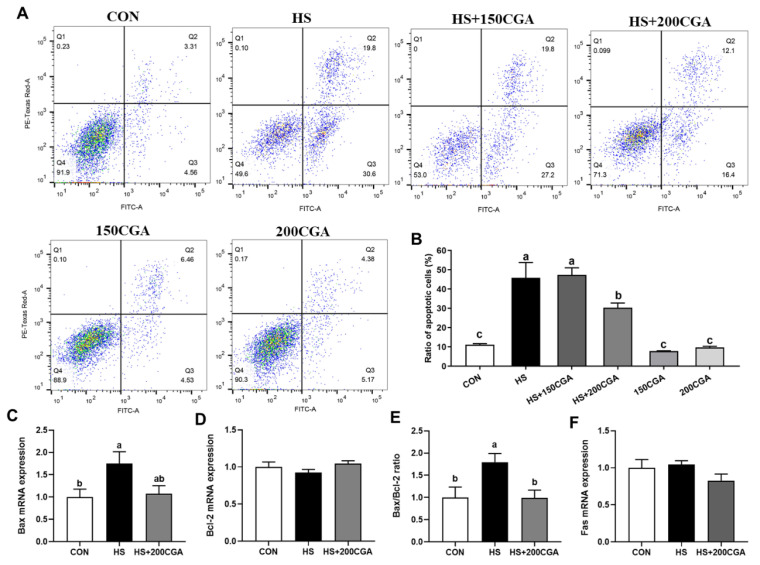
Effects of chlorogenic acid (CGA) on cell apoptosis of rabbit IECs. (**A**,**B**) Cell apoptosis analyzed by flow cytometric assays. In each diagram, Q1, Q2, Q3 and Q4 represent the percentages of nonviable and necrotic cells, late apoptotic cells, early apoptotic cells, and live cells, respectively. (**C**–**F**) The expression of apoptosis-related genes. *Bax*, Bcl-2-associated X protein; *Bcl-2*, B-cell lymphoma 2. CON, untreated control; HS, cells exposed to heat stress (HS) for 6 h; HS+200 CGA, cells treated with 200 μM CGA and exposed to HS for 6 h. ^a,b,c^ Means with different letters are significantly different (*p* < 0.05).

To investigate the effect of CGA on HS-induced apoptosis, flow cytometry was employed to detect cell apoptosis. As shown in Figure 4A,B, the proportion of apoptotic cells was significantly increased (*p* < 0.05) in the HS group compared to the CON group. However, when CGA was administered at a concentration of 200 μM, the proportion of apoptotic cells was significantly reduced (*p* < 0.05) relative to the HS group. Notably, the 200 CGA group alone showed a low proportion of apoptotic cells, similar to the CON group. However, 150 μM CGA did not have any alleviating effect on HS-induced apoptosis. Therefore, 200 μM CGA was used in subsequent experiments. Consistently, the mRNA expression levels of apoptosis-related genes (Figure 4C–F) showed that *Bax* expression and the *Bax*/*Bcl-2* ratio were elevated (*p* < 0.05) in the HS group compared to the CON group, while both parameters were significantly lower (*p* < 0.05) in the HS+200 μM CGA group than in the HS group. No significant differences were observed in the mRNA expression levels of *Bcl-2* or *Fas* among the CON, HS, and HS+200 μM CGA groups.

### 3.5. CGA Enhances the Antioxidant Capacity in HS-Challenged Rabbit IECs (In Vitro)

**Figure 5:**
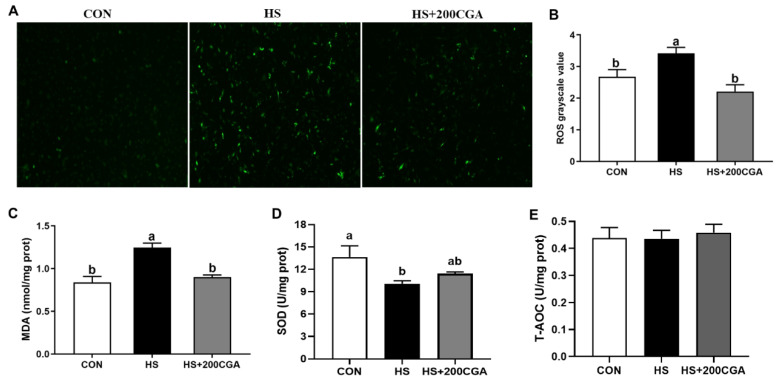
Chlorogenic acid (CGA) enhances the antioxidant capacity in heat stress (HS)-challenged rabbit intestinal epithelial cells. (**A**,**B**) Intracellular ROS levels assessed by immunofluorescence staining. Microscope’s magnification is 100x. (**C**–**E**) Intracellular malondialdehyde (MDA), superoxide dismutase (SOD), and total antioxidant capacity (T-AOC) levels. CON, untreated control; HS, cells exposed to heat stress (HS) for 6 h; HS+200 CGA, cells treated with 200 μM CGA and exposed to HS for 6 h. ^a,b^ Means with different letters are significantly different (*p* < 0.05).

Immunofluorescence analysis showed that HS induced (*p* < 0.05) the production of cellular ROS (Figure 5). In addition, HS increased (*p* < 0.05) the content of cellular MDA and decreased (*p* < 0.05) the activity of SOD. However, CGA can alleviate HS-induced oxidative stress by decreasing MDA and ROS concentrations and increasing SOD activity (*p* < 0.05), while having no significant effect on T-AOC (*p* > 0.05).

### 3.6. CGA Promotes the Expression of Tight Junction-Related Protein in HS-Challenged Rabbit IECs (In Vitro)

**Figure 6:**
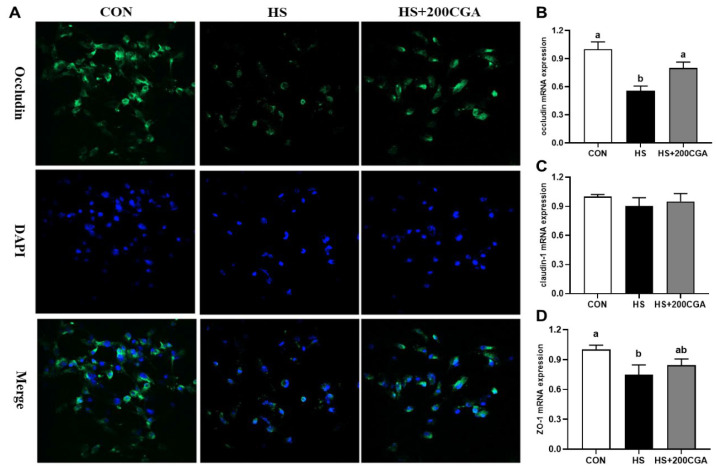
Chlorogenic acid (CGA) promotes the expression of tight junction proteins expressions in heat stress (HS)-challenged rabbit IECs. (**A**) Localization of the tight-junction protein occludin by immunofluorescence staining. Microscope’s magnification is 100x. (**B**–**D**) qPCR analysis of the expression levels of tight junction proteins. *ZO-1*, Zonula occludens 1. CON, untreated control; HS, cells exposed to heat stress (HS) for 6 h; HS+200 CGA, cells treated with 200 μM CGA and exposed to HS for 6 h. ^a,b^ Means with different letters are significantly different (*p* < 0.05).

As shown in Figure 6, HS reduced (*p* < 0.05) the fluorescence intensity of occludin, a key TJP, whereas pretreatment with 200 μM CGA remarkably restored (*p* < 0.05) occludin expression. At the transcriptional level, HS downregulated the occludin and ZO-1 mRNA expression (*p* < 0.05). However, CGA reversed (*p* < 0.05) the HS-induced suppression of these two TJPs. There were no significant differences in claudin-1 mRNA expression among the treatment groups (*p* > 0.05).

### 3.7. CGA Promotes the Activation of Nrf2 Signaling in HS-Challenged Rabbit IECs (In Vitro)

**Figure 7:**
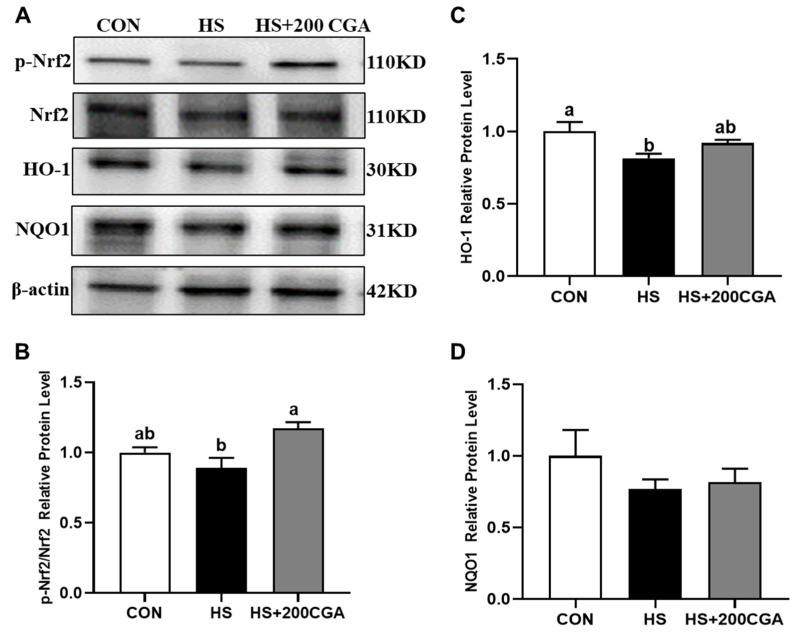
Chlorogenic acid (CGA) promotes the activation of Nrf2/HO-1 signaling in heat stress (HS)-challenged rabbit IECs. (**A**) Western blot analysis of key proteins in the Nrf2 signaling. (**B**–**D**) Grayscale value analysis of protein bands. Nrf2, nuclear factor erythroid-derived-related factor 2; HO-1, Heme-oxygenase 1; NQO1, NAD(P)H quinone oxidoreductase-1. CON, untreated control; HS, cells exposed to heat stress (HS) for 6 h; HS+200 CGA, cells treated with 200 μM CGA and exposed to HS for 6 h. ^a,b^ Means with different letters are significantly different (*p* < 0.05).

HS suppressed the activation of the Nrf2 signaling, as evidenced by decreased (*p* < 0.05) levels of p-Nrf2 and HO-1 (Figure 7). In contrast, 200 μM CGA activated (*p* < 0.05) Nrf2 and partially restored HO-1 expression. The NQO1 level did not differ significantly among all experimental groups (*p* > 0.05).

### 3.8. CGA Attenuates HS-Induced Oxidative Damage in Rabbit IECs via Nrf2 Signaling (In Vitro)

**Figure 8:**
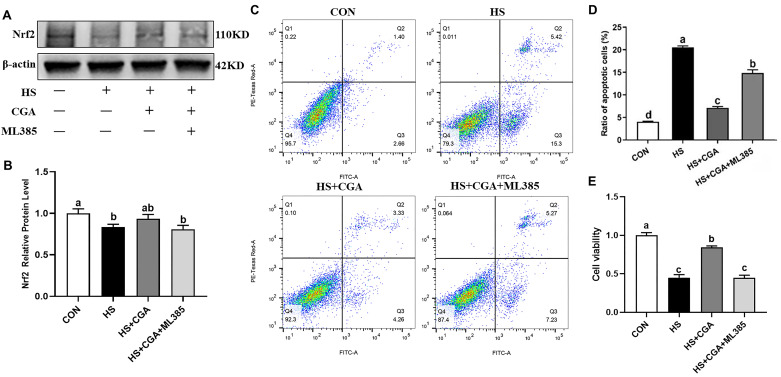
Chlorogenic acid (CGA) attenuates heat stress (HS)-induced injury in rabbit IECs via nuclear factor erythroid-derived-related factor 2 (Nrf2) signaling. (**A**) Nrf2 protein analysis by Western blot. (**B**) Grayscale value analysis of protein bands. (**C**,**D**) In each diagram, Q1, Q2, Q3 and Q4 represent the percentages of nonviable and necrotic cells, late apoptotic cells, early apoptotic cells, and live cells, respectively. (**E**) Cell viability. CON, untreated control; HS, cells exposed to heat stress (HS) for 6 h; HS+ CGA, cells treated with 200 μM CGA and exposed to HS for 6 h. ^a,b,c,d^ Means with different letters are significantly different (*p* < 0.05).

**Figure 9:**
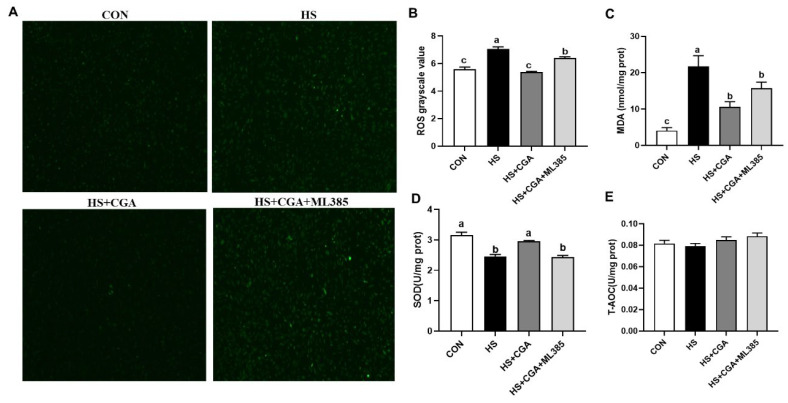
Inhibition of nuclear factor erythroid-derived-related factor 2 (Nrf2) activity blocks the antioxidant effect of chlorogenic acid (CGA). (**A**,**B**) Intracellular ROS levels assessed by immunofluorescence staining. Microscope’s magnification is 100x. (**C**) Intracellular malondialdehyde (MDA) levels. (**D**,**E**) Intracellular contents of superoxide dismutase (SOD) and total antioxidant capacity (T-AOC). CON, untreated control; HS, cells exposed to heat stress (HS) for 6 h; HS+ CGA, cells treated with 200 μM CGA and exposed to HS for 6 h. ^a,b,c^ Means with different letters are significantly different (*p* < 0.05).

To further explore the role of Nrf2 activation in the protective effects of CGA against HS-induced cellular oxidative damage, a specific Nrf2 inhibitor (ML385) was employed in this study. Our findings show that CGA significantly mitigated (*p* < 0.05) HS-induced down-regulation of Nrf2 (Figure 8). The Nrf2 inhibitor ML385 not only abolished this protective effect but also negated the antioxidant capacity of CGA, as demonstrated by significantly reduced cell viability, elevated apoptosis, and increased production of MDA and ROS (*p* < 0.05), as well as decreased (*p* < 0.05) SOD activity in HS-exposed cells (Figure 9).

## 4. Discussion

HS has become a major global environmental stressor, causing substantial economic losses to the rabbit industry [19]. Several studies have elucidated that exposure to HS disrupts redox balance, leading to oxidative stress in rabbits [4,20,21]. The results of the present study indicated that HS induced systemic oxidative stress in rabbits, as evidenced by the significant reduction in SOD and CAT activities and the elevation of serum MDA concentration. CAT and SOD are important antioxidant enzymes constituting the primary cellular defense against oxidative injury [22], while MDA is commonly regarded as an index of lipid peroxidation [23]. In this study, dietary CGA supplementation reversed these changes, indicating its ability to enhance systemic antioxidant capacity. The protective effect is likely partly attributed to CGA’s polyphenolic structure, characterized by multiple hydroxyl groups, which enables it to scavenge free radicals directly and modulate the transcription of antioxidant enzyme genes [24].

The intestine is highly susceptible to HS due to its high metabolic activity and direct exposure to external stimuli [25]. Under HS conditions, excessive reactive oxygen species (ROS) production disrupts the redox balance, triggering lipid peroxidation and impairing antioxidant defense systems [26,27]. Heat shock protein 70 (HSP70) is an important member of the heat shock protein family and plays an important role in resisting oxidative damage [28,29]. Under normal conditions, it maintains low expression levels but is strongly induced upon exposure to HS or other stimuli [30]. Therefore, HSP70 is considered as an ideal biomarker for assessing the level of HS in animals [31]. In the present study, HS-induced upregulation of jejunal HSP70 was suppressed by CGA, confirming the protective role of CGA in reducing HS-related cellular stress and intestinal damage. Oxidative stress is the primary pathological trigger in HS-induced intestinal injury [32]. It directly induces cellular apoptosis, while concurrently damaging the intestinal barrier; both processes converge to compromise intestinal homeostasis [7,33]. Excessive ROS not only triggers apoptotic signaling cascades but also degrades the expression of tight junction proteins, leading to increased intestinal permeability [34]. In the present study, HS downregulated the expression of antioxidant genes (*HO-1*, *SOD1* and *GPx1*) and upregulated pro-apoptotic genes (*Bax* and *caspase-3*) in the jejunum of rabbits, while CGA effectively reversed these alterations, which was consistent with a previous study in IECs [35]. These findings demonstrate that CGA alleviates HS-induced intestinal oxidative damage in rabbits.

The intestinal epithelial barrier consists of IECs and intercellular junctions. Tight junctions serve as the primary form of these cellular connections and are critically associated with the integrity of the intestinal barrier [36]. At the cellular level, HS promoted rabbit IEC apoptosis, increased ROS production, and reduced the expression of tight junction proteins (*occludin*, *ZO-1*)—key components that form the physical barrier to prevent luminal toxin translocation [37]. CGA supplementation alleviated these adverse effects, suggesting that its protective action on the structural integrity of intestinal tight junctions involves both suppressing oxidative stress-induced apoptosis and preserving tight junction structure. This is consistent with previous findings that CGA improves intestinal barrier function in weaned pigs by enhancing antioxidant capacity and reducing inflammation [36], highlighting its conserved protective effects across livestock species.

The nuclear factor erythroid-derived-related factor 2 (Nrf2) signaling pathway is one of the central regulators of cellular antioxidant defense, as it translocates to the nucleus upon activation and binds to antioxidant response elements (AREs) to orchestrate the expression of numerous antioxidant and detoxifying enzymes (e.g., HO-1, SOD1) [38,39]. The present study revealed that HS suppressed Nrf2 activation, as evidenced by reduced levels of p-Nrf2 and its downstream target HO-1 in IECs. This is in line with the previous report that HS inhibits Nrf2 signaling by promoting its ubiquitination and degradation, thereby weakening cellular antioxidant defense [40]. CGA treatment effectively restored Nrf2 activation, which was associated with improved antioxidant capacity and reduced apoptosis, indicating that CGA may maintain intestine barrier integrity by activating Nrf2 signaling.

To further explore the molecular mechanism underlying the antioxidant effects of CGA, we used the Nrf2-specific inhibitor ML385 (a small-molecule inhibitor that blocks Nrf2-ARE binding [41]), which abolished CGA’s protective effects, including the restoration of cell viability, reduction in ROS and MDA production, and enhancement of SOD activity. This mechanistic evidence directly indicates that CGA exerts its antioxidant and anti-apoptotic effects through activating the Nrf2 signaling pathway. The upstream triggers of Nrf2 activation by CGA may involve direct interaction with Nrf2 regulatory proteins (e.g., Keap1, which retains Nrf2 in the cytoplasm under normal conditions [42]) or indirect modulation via ROS scavenging [43], though further studies are needed to clarify these molecular interactions.

## 5. Conclusions

This study demonstrates that CGA activates Nrf2 signaling, which enhances antioxidant enzyme capacity (SOD and CAT) to scavenge excessive ROS, suppresses cellular apoptosis, and preserves the expression of tight junction proteins (*occludin*, *ZO-1*) to maintain intestinal barrier integrity in rabbits. These findings not only deepen our understanding of CGA’s antioxidant mechanisms in the context of HS but also provide a scientific basis for its application as a safe and effective nutritional supplement to improve HS resistance in rabbits.

## Figures and Tables

**Table 1:** Impacts of chlorogenic acid (CGA) on the serum redox status of rabbits under heat stress (HS) ^2^.

Items ^1^	CON	HS	HS+CGA	SEM	*p*-Values
MDA (nmol/mL)	1.64 ^b^	2.53 ^a^	1.80 ^b^	0.087	<0.001
CAT (U/mL)	3.46 ^a^	1.98 ^b^	3.12 ^a^	0.197	0.036
GSH-Px (U/mL)	201.64	211.29	210.86	2.992	0.126
SOD (U/mL)	74.21 ^a^	59.37 ^b^	79.03 ^a^	2.363	<0.001
T-AOC (U/mL)	0.91	0.82	0.83	0.021	0.371

^1^ MDA, malondialdehyde; CAT, catalase; GSH-Px, glutathione peroxidase; SOD, superoxide dismutase; T-AOC, total antioxidant capacity. ^2^ CON, rabbits fed a basal diet and housed at optimum temperature; HS, rabbits fed a basal diet and exposed to HS; HS+CGA, rabbits fed a basal diet supplemented with 800 mg/kg CGA and exposed to HS. ^a,b^ means significant difference (*p* < 0.05) between treatment groups. n = 8.

## Data Availability

The original contributions presented in this study are included in the article/Supplementary Materials. Further inquiries can be directed to the corresponding author.

## Supplementary Materials

The following supporting information can be downloaded at: https://www.mdpi.com/article/10.3390/antiox15010002/s1, Table S1: Ingredient composition and nutrient levels of basal diet (as-fed basis); Table S2: Primers used for real-time quantitative PCR.
