# Supplementary material for: Dietary Chlorogenic Acid Supplementation Alleviates Heat Stress-Induced Intestinal Oxidative Damage by Activating Nrf2 Signaling in Rabbits
Source: Antioxidants (Basel). 2025 Dec 19;15(1):2. doi: 10.3390/antiox15010002 (PMC12837396; doi:10.3390/antiox15010002)
Supplement: Supplementary file 1 [file antioxidants-15-00002-s001.zip › antioxidants-3988524-supplementary.pdf]

Table S1. Ingredient composition and nutrient levels of basal diet (as-fed basis)

Items	Content (%)
Ingredients	
Corn	17.00
Peanut seedling	17.00
Alfalfa meal	25.40
beanstalk	5.00
Soybean oil	0.60
Soybean meal	17.00
Wheat bran	14.00
Premix[1)]	4.00
Total	100
Calculated nutrient levels	
DE (MJ/kg)	9.73
CP	15.69
EE	3.19
CF	17.12

[1)] The premix provided the following per kg of diets: Vitamin D3, 2,000 IU; Vitamin B5, 375 mg; Vitamin E, 22.5 mg; Vitamin A, 6,000 IU; Vitamin K, 15 mg; Vitamin B2, 45 mg; Vitamin B1, 15 mg; Vitamin B6, 15 mg; Vitamin B7, 1.5 mg; Vitamin B3, 375 mg; Vitamin B12, 0.15 mg; Ferrous sulfate, 100 mg; Choline chloride, 625 mg; Selenium, 0.05 mg; Iodine, 0.6 mg; Zinc, 50 mg; Methionine, 1,500 mg; Iysine, 1,500 mg.

Table S2. Primers used for real-time quantitative PCR[1]

Genes	Accession NO.	Primer sequences[2] (5′-3′)	Size, bp
GAPDH	NM_001082253.1	F:TGCCACCCACTCCTCTACCTTCG<br>R:CCGGTGGTTTGAGGGCTCTTACT	163
GPX1	NM_001085444.1	F:CAGGAGAACGCCAAGAATGAGGAG<br>R:GTTCACCTCGCACTTCTGGAAGAG	105
NQO1	XM_002711667.4	F:AGCGGCTCCATGTACTCTCTCC<br>R:GAGTGTGCCCGATGCTGTATGTG	136
SOD1	NM_001082627.2	F:CGCATAACAGGACTGACCGAAGG<br>R:ATTAACACATCAGCCACACCATTGC	197
SOD2	XM_051854201.1	F:TTTCTGGACAAACCTGAGCCCTAAC<br>R:CCGTCAGCCTCTCCTTGAACTTG	110
HO-1	XM_051846030.1	F:CCACCAAGTTCAAGCAGCTCTACC<br>R:TTAGCCTCTTCCACCACCCTCTG	88
Caspase-3	NM_001354777.2	F:CTAAGCCACGGTGATGAAGGAGTC<br>R:CACTGTCTGTCTCGATGCCACTG	175
Fas	NM_001081995.1	F:TCTGGGTACTGCTGCCTCTGATAC<br>R:ACAGTCTGGTTTGCCCTCATTGC	191
Bax	XM_002723696.4	F:TATGGGCTGGACGCTGGACTTC<br>R:AGATGGTGAGTGAGGCGGTGAG	155
Bcl-2	XM_008261439.3	F:GTTCGGTGGGGTCATGTGTGTG<br>R:AGGTGCCGGTTCAGGTACTCAG	99
Occludin	XM_008262318.3	F:CTTGCCTGGGACAGAACCTA<br>R:AGCCATAACCGTAGCCGTAA	121
Claudin-1	NM_001089316.1	F:GGAGCAAAAGATGCGGATGG<br>R:AATTGACAGGGGTCAAAGGGT	93

ZO-1	XM_008269782.1	F: GACTGATGCGAAGACGTTGA<br>R: GCAGAATGGATGCTGTCAGA	117

[1]GPX1: Glutathione peroxidase 1, NQO1: NAD(P)H dehydrogenase quinone 1, SOD: superoxide dismutase, HO-1: Hemeoxygenase-1, Bax: B-cell lymphoma-2-associated X protein, Bcl-2: B-cell lymphoma-2, ZO-1: Zonula occludens 1.

[2]F: forward, R: reverse.
